# Development of a selective agonist for relaxin family peptide receptor 3

**DOI:** 10.1038/s41598-017-03465-7

**Published:** 2017-06-12

**Authors:** Dian Wei, Meng-Jun Hu, Xiao-Xia Shao, Jia-Hui Wang, Wei-Han Nie, Ya-Li Liu, Zeng-Guang Xu, Zhan-Yun Guo

**Affiliations:** Research Centre for Translational Medicine at East Hospital, College of Life Sciences and Technology, Tongji University, Shanghai, China

## Abstract

Relaxin family peptides perform a variety of biological functions by activating four G protein-coupled receptors, namely RXFP1–4. Among these receptors, RXFP3 lacks a specific natural or synthetic agonist at present. A previously designed chimeric R3/I5 peptide, consisting of the B-chain of relaxin-3 and the A-chain of INSL5, displays equal activity towards the homologous RXFP3 and RXFP4. To increase its selectivity towards RXFP3, in the present study we conducted extensive mutagenesis around the B-chain C-terminal region of R3/I5. Decreasing or increasing the peptide length around the B23–B25 position dramatically lowered the activation potency of R3/I5 towards both RXFP3 and RXFP4. Substitution of B23Gly with Ala or Ser converted R3/I5 from an efficient agonist to a strong antagonist for RXFP3, but the mutants retained considerable activation potency towards RXFP4. Substitution of B24Gly increased the selectivity of R3/I5 towards RXFP3 over the homologous RXFP4. The best mutant, [G(B24)S]R3/I5, displayed 20-fold higher activation potency towards RXFP3 than towards RXFP4, meanwhile retained full activation potency at RXFP3. Thus, [G(B24)S]R3/I5 is the best RXFP3-selective agonist known to date. It is a valuable tool for investigating the physiological functions of RXFP3, and also a suitable template for developing RXFP3-specific agonists in future.

## Introduction

Relaxin family is a group of peptide hormones, including relaxin (primates have two relaxin genes), relaxin-3, and insulin-like peptide 3–6 (INSL3-6)^1–5^. These peptides perform a variety of biological functions^1–5^, such as regulating reproduction, food intake, stress responses, and glucose homeostasis. To date, four formerly orphan G protein-coupled receptors (GPCRs) have been identified as their receptors and renamed relaxin family peptide receptor 1–4 (RXFP1–4). Relaxin and INSL3 are the cognate agonists of the homologous RXFP1 and RXFP2, respectively^6, 7^. Relaxin-3 and INSL5 are the cognate agonists of the homologous RXFP3 and RXFP4, respectively^8, 9^. In addition, relaxin-3 can also activate RXFP1 and RXFP4 *in vitro* with high efficiency^10, 11^.

Among these receptors, the homologous RXFP1 and RXFP2 belong to the leucine-rich repeat (LRR)-containing GPCR subfamily, and both contain a large extracellular N-terminal domain with 10 LRRs and a unique N-terminal low-density lipoprotein receptor type A (LDLa) module. The LRR module forms the high affinity ligand-binding site that primarily interacts with the essential B-chain residues of their respective ligand^12–15^, and the LDLa module is critical for receptor activation^16–19^. The extracellular loops form a low affinity ligand-binding site that primarily interacts with the A-chain residues of their respective ligand^10, 20–23^. By contrast, the homologous RXFP3 and RXFP4 are classical peptide receptors, with a short N-terminal domain, and thus, their extracellular loops form the primary ligand-binding site. Indeed, recent studies suggest that a highly conserved WxxExxxD motif at the extracellular end of the second transmembrane domain of RXFP3 and RXFP4 plays a critical role by forming electrostatic and hydrophobic interactions with the positively charged B-chain Arg residues and the large aromatic B-chain C-terminal Trp residue of the ligand^24–29^.

RXFP3 and its cognate agonist relaxin-3 are primarily expressed in the brain and this receptor–ligand pair is implicated in the regulation of food intake, stress responses, arousal and exploratory behaviors^30–34^. However, RXFP3 lacks a specific natural or synthetic agonist at present. Its cognate agonist relaxin-3 can also efficiently activate RXFP1 and RXFP4. A previously designed chimeric R3/I5 peptide, comprising the B-chain of relaxin-3 and the A-chain of INSL5, is not able to activate RXFP1, but activate RXFP3 and RXFP4 with almost equal potency^35^. Some stapled relaxin-3 B-chain analogues and some simplified relaxin-3 analogues display higher selectivity (5–30-fold) for RXFP3 than for the homologous RXFP4, but their activity towards RXFP3 is much lower than that of wild-type relaxin-3 or R3/I5^36, 37^. Previous studies disclosed that the B-chain C-terminal B26Arg and B27Trp are essential for relaxin-3 or R3/I5 to activate receptor RXFP3 and RXFP4^24–29^. Our recent study revealed that changing the B-chain C-terminal conformation of R3/I5 can selectively abolish its activity towards RXFP3^38^. Thus, we speculated that engineering of the B-chain C-terminal region of R3/I5 might increase its selectivity towards RXFP3.

**Figure 1:**
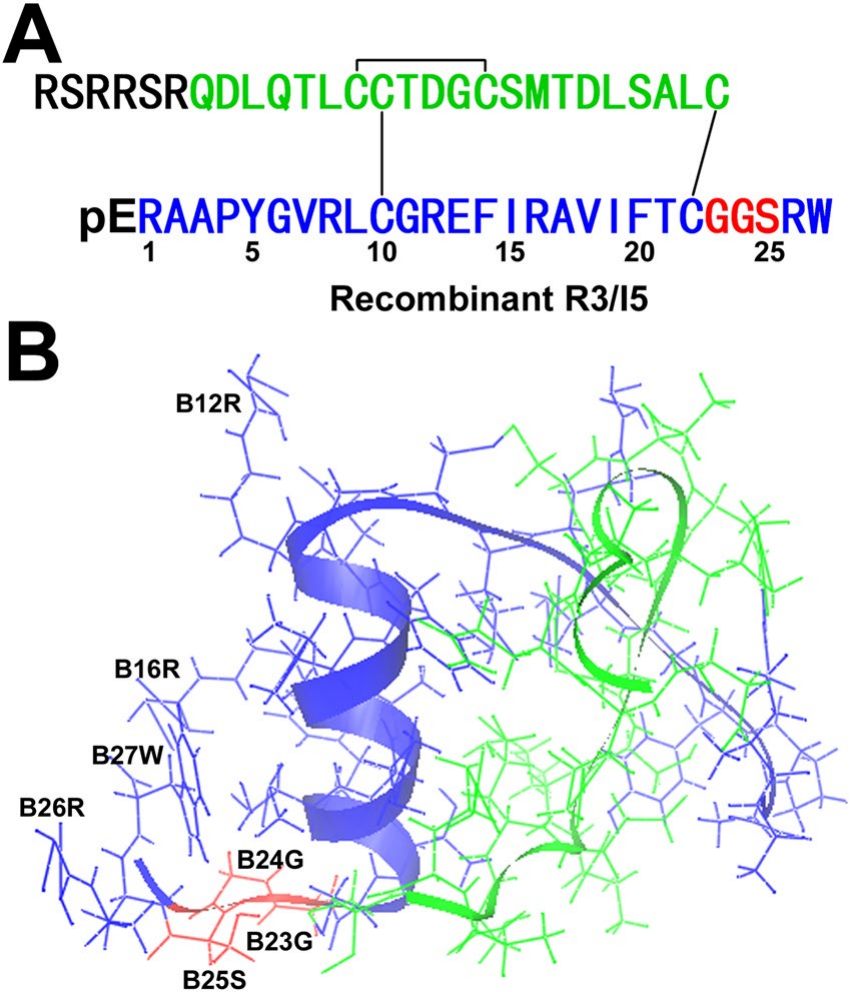
Amino acid sequence and three-dimensional structure of the chimeric R3/I5 peptide. (**A**) Amino acid sequence and disulfide linkages of the recombinant R3/I5 peptide. Disulphide linkages are shown as lines. For the B-chain, the B23–B25 position is shown in red, the introduced N-terminal pyroglutamate residue (pE) in black, and other parts in blue. For the A-chain, the N-terminal solubilising tag is shown in black, and other parts in green. (**B**) The previously reported solution structure of R3/I5 (PDB code 2K1V)^39^. The A-chain is shown in green and the B-chain in blue, except the B23–B25 position in red.

As shown in Fig. 1, the two-chain R3/I5 peptide folds into a globular structure similar to that of relaxin-3^39, 40^: Both have a folded-back B-chain C-terminus presumably due to presence of two highly conserved Gly residues at the B23 and B24 positions. By contrast, the B-chain C-terminus of INSL5 adopts an extended α-helical conformation because its corresponding positions are typically occupied by larger Ala and Ser residues^41^. To generate an agonist with higher selectivity for RXFP3, in the present study we conducted extensive mutagenesis around the B-chain C-terminal region of the chimeric R3/I5 peptide. On one hand, we changed the peptide length around the B23–B25 region by deleting some residues or inserting an additional Gly residue. Insertion or deletion in this region likely affects the position and orientation of the important B26Arg and B27Trp residues, and thus might affect the receptor selectivity of R3/I5. On the other hand, we substituted the highly conserved B23Gly, B24Gly, and B25Ser residues with some other amino acids. These substitutions likely affect the flexibility of the B-chain C-terminus and thus might modulate receptor selectivity of R3/I5. Using these approaches, we obtained a selective and fully active agonist for RXFP3, [G(B24)S]R3/I5, that displayed 20-fold higher activation potency towards RXFP3 than towards RXFP4, and retained full activity at RXFP3 compared with wild-type R3/I5. To our knowledge, [G(B24)S]R3/I5 is the best RXFP3-selective agonist known to date.

## Results

### Preparation of R3/I5 mutants

**Figure 2:**
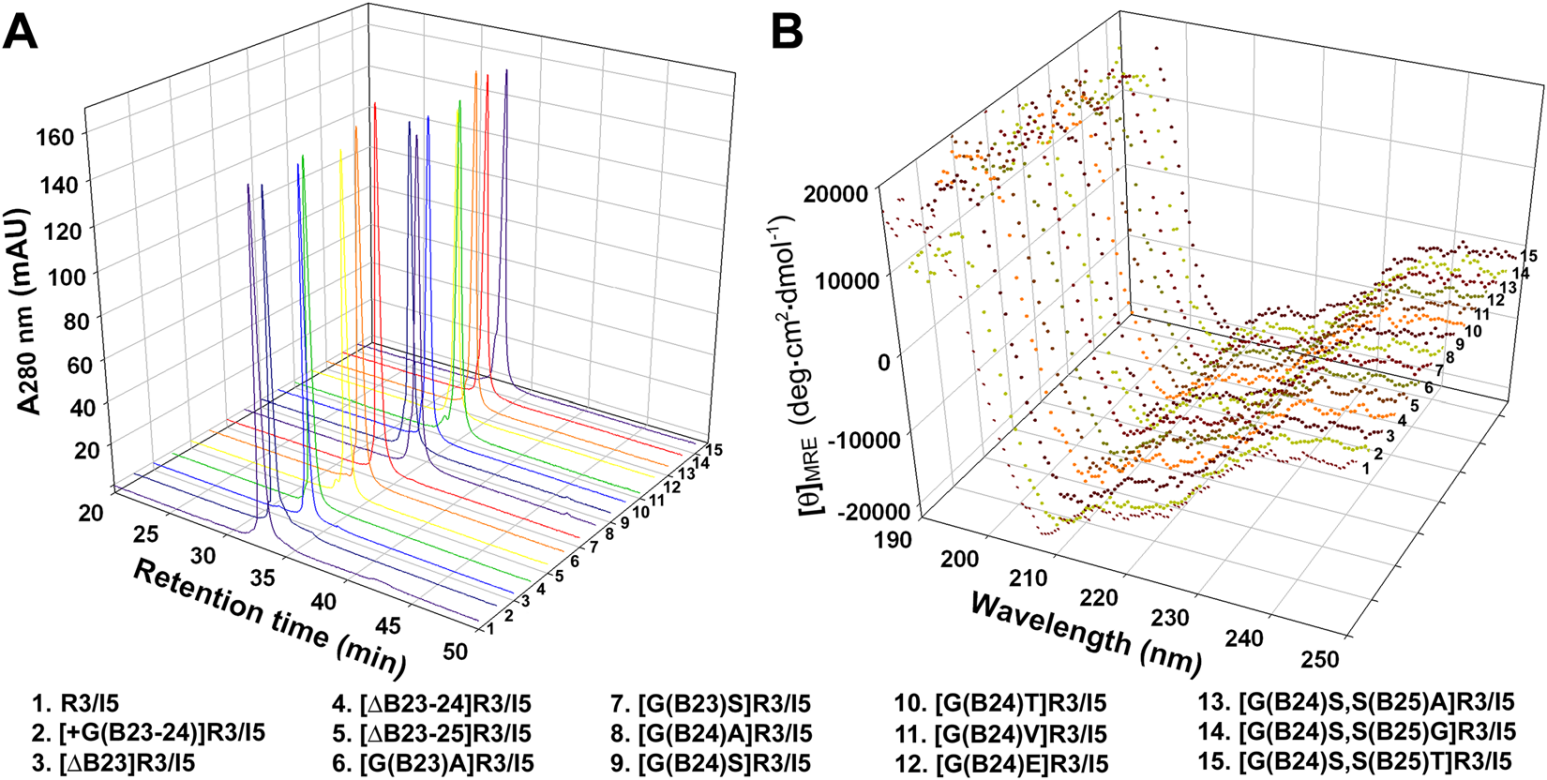
Characterisation of the mature R3/I5 mutants. (**A**) Purity analysis of the mature R3/I5 mutants by C18 reverse-phase HPLC. (**B**) Structural analysis of the mature R3/I5 mutants by circular dichroism.

In the present study, we conducted extensive mutagenesis around the B-chain C-terminal region of R3/I5 and generated 14 mutants. All mutants were overexpressed in *Escherichia coli* as a single-chain precursor according to our previously reported procedure^38, 42^. After solubilisation from inclusion bodies using an S-sulfonation approach, mutant precursors were purified by immobilized metal ion affinity chromatography and subjected to in vitro refolding. Thereafter, refolded precursors were purified by high performance liquid chromatography (HPLC) and sequentially treated with endoproteinase Lys-C, papaya glutaminyl cyclase, and carboxypeptidase B, according to our previous procedure^38, 42^. The resultant mature mutants were further purified by HPLC and their identities were confirmed by mass spectrometry. All mutants displayed the expected molecular masses, indicating the presence of the expected mutation and the correct processing of the mutant precursors (Supplementary Table S1). Purity of these mutants was confirmed by HPLC analysis using an analytical C18 reverse-phase column (Fig. 2A): all displayed a single symmetric peak, indicating their homogeneity. The secondary structure of these mutants was analysed by circular dichroism spectroscopy (Fig. 2B): their spectra were similar to that of the template, suggesting that mutation did not disturb the overall structure of R3/I5.

### Insertion or deletion in B23–B25 region affects the activity of R3/I5 towards RXFP3 and RXFP4

**Figure 3:**
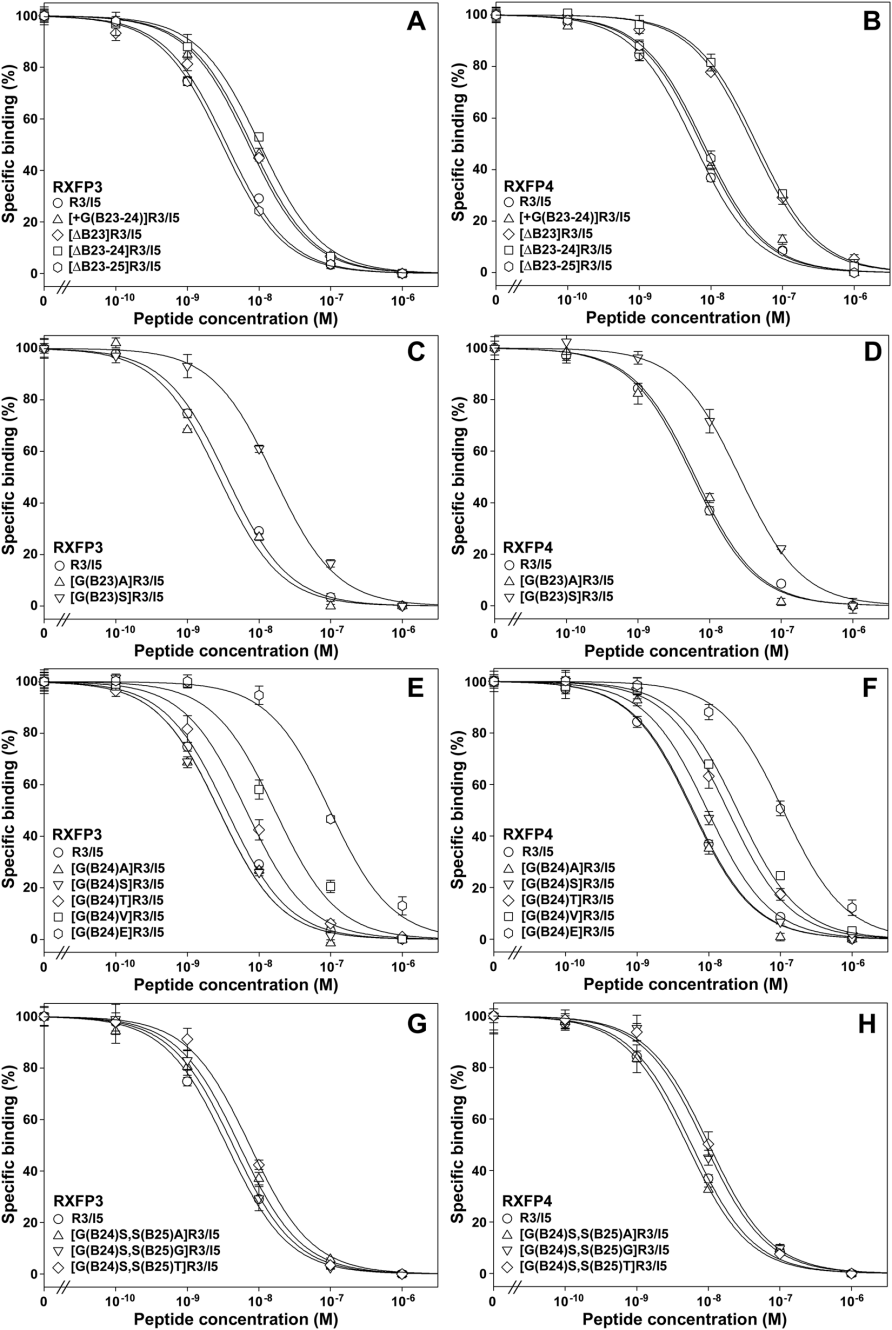
Binding potency of the mature R3/I5 mutants towards RXFP3 and RXFP4. A NanoLuc-conjugated R3/I5 peptide was used as the tracer and HEK293T cells transiently overexpressing human RXFP3 or human RXFP4 were used as the receptor source. Nonspecific binding was determined by competition with 1.0 μM R3/I5. Specific binding data are expressed as means ± SE (*n* = 3) and fitted to sigmoidal curves using SigmaPlot 10.0 software. The calculated pIC_50_ values are summarized in Table 1. Data are representative of at least two independent assays that gave essentially same results.

**Figure 4:**
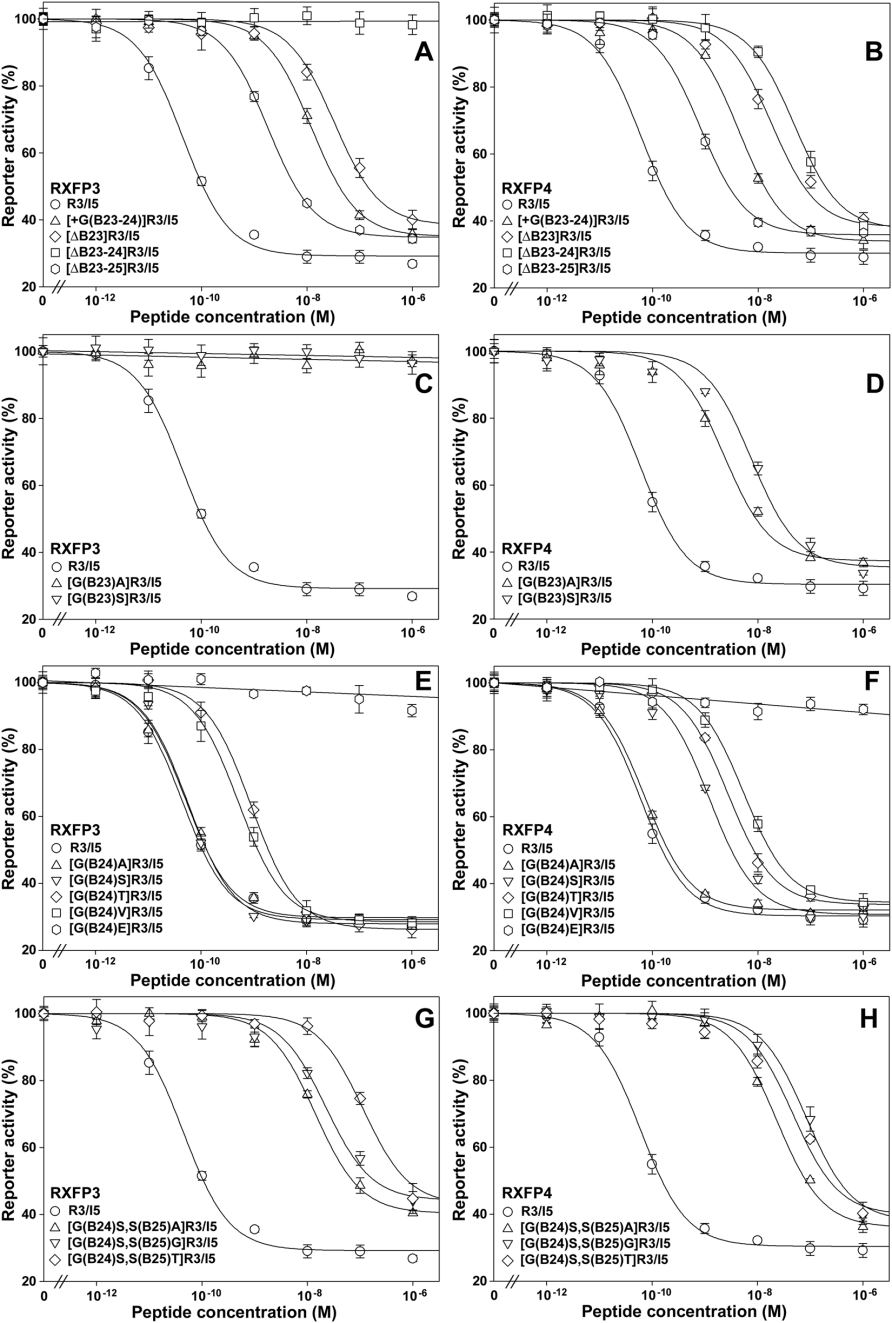
Activation potency of the mature R3/I5 mutants towards RXFP3 and RXFP4. HEK293T cells transiently cotransfected with a CRE-controlled NanoLuc reporter vector and the expression construct of human RXFP3 or human RXFP4 were used for activation assays. The measured bioluminescence data are expressed as means ± SE (*n* = 3) and fitted to sigmoidal or linear curves using SigmaPlot 10.0. The calculated pEC_50_ values are summarized in Table 1. Data are representative of at least two independent assays that gave essentially same results.

To modulate the selectivity of R3/I5 towards RXFP3 and RXFP4, we first tried changing the peptide length around the B23–B25 position. This region is highly conserved as Gly-Gly-Ser from mammals, birds, reptiles, to fishes (Supplementary Fig. S1). To increase the peptide length, we inserted a Gly residue between B23 and B24. Unfortunately, the resultant mutant, designated [+G(B23–24)]R3/I5, displayed ~100-fold lower activation potency towards both RXFP3 and RXFP4, although the binding potency for both receptors was comparable with R3/I5 (Figs 3A,B and 4A,B and Table 1). Thus, increasing the length at this position had a serious detrimental effect on the activity of R3/I5 towards both RXFP3 and RXFP4.

**Table 1:** Summary of the measured pIC_50_ and pEC_50_ values of the mature R3/I5 mutants towards human RXFP3 and human RXFP4.

Peptides	RXFP3		RXFP4	
	pIC_50_ (IC_50_)	pEC_50_ (EC_50_)	pIC_50_ (IC_50_)	pEC_50_ (EC_50_)
R3/I5	8.45 ± 0.03 (3.54 ± 0.26)	10.35 ± 0.05 (0.045 ± 0.005)	8.23 ± 0.03 (5.88 ± 0.42)	10.22 ± 0.05 (0.060 ± 0.008)
[+G(B23-24)]R3/I5	8.10 ± 0.03 (7.94 ± 0.57)	7.92 ± 0.04 (12.02 ± 1.16)	8.14 ± 0.04 (7.24 ± 0.70)	8.37 ± 0.06 (4.26 ± 0.63)
[∆B23]R3/I5	8.15 ± 0.04 (7.07 ± 0.69)	7.49 ± 0.07 (32.35 ± 5.66)	7.42 ± 0.03 (38.01 ± 2.72)	7.75 ± 0.07 (17.78 ± 3.11)
[∆B23-24]R3/I5	7.99 ± 0.04 (10.23 ± 0.99)	N.D. (N.D.)	7.36 ± 0.03 (43.65 ± 3.12)	7.31 ± 0.06 (48.97 ± 7.26)
[∆B23-25]R3/I5	8.51 ± 0.02 (3.09 ± 0.14)	8.74 ± 0.04 (1.81 ± 0.18)	8.10 ± 0.02 (7.94 ± 0.37)	9.10 ± 0.05 (0.79 ± 0.10)
[G(B23)A]R3/I5	8.56 ± 0.04 (2.75 ± 0.26)	N.D. (N.D.)	8.20 ± 0.05 (6.30 ± 0.77)	8.64 ± 0.07 (2.29 ± 0.40)
[G(B23)S]R3/I5	7.79 ± 0.04 (16.21 ± 1.57)	N.D. (N.D.)	7.58 ± 0.06 (26.30 ± 3.89)	8.12 ± 0.06 (7.58 ± 1.12)
[G(B24)A]R3/I5	8.56 ± 0.04 (2.75 ± 0.26)	10.29 ± 0.04 (0.051 ± 0.005)	8.22 ± 0.04 (6.02 ± 0.58)	10.14 ± 0.04 (0.072 ± 0.007)
[G(B24)S]R3/I5	8.56 ± 0.04 (2.75 ± 0.26)	10.25 ± 0.04 (0.056 ± 0.006)	8.02 ± 0.04 (9.54 ± 0.93)	8.92 ± 0.05 (1.20 ± 0.14)
[G(B24)V]R3/I5	7.78 ± 0.06 (16.59 ± 2.46)	9.29 ± 0.06 (0.51 ± 0.08)	7.60 ± 0.04 (25.11 ± 2.43)	8.27 ± 0.05 (5.37 ± 0.65)
[G(B24)T]R3/I5	8.18 ± 0.05 (6.60 ± 0.81)	9.05 ± 0.05 (0.89 ± 0.11)	7.74 ± 0.05 (18.19 ± 2.22)	8.57 ± 0.05 (2.69 ± 0.32)
[G(B24)E]R3/I5	7.01 ± 0.05 (97.72 ± 10.63)	N.D. (N.D.)	6.96 ± 0.05 (109.6 ± 13.4)	N.D. (N.D.)
[G(B24)S,S(B25)A]R3/I5	8.27 ± 0.04 (5.37 ± 0.51)	7.85 ± 0.05 (14.12 ± 1.72)	8.30 ± 0.06 (5.01 ± 0.74)	7.63 ± 0.07 (23.44 ± 4.10)
[G(B24)S,S(B25)G]R3/I5	8.36 ± 0.05 (4.36 ± 0.53)	7.65 ± 0.10 (22.38 ± 5.80)	8.05 ± 0.06 (8.91 ± 1.32)	7.08 ± 0.05 (83.17 ± 10.15)
[G(B24)S,S(B25)T]R3/I5	8.13 ± 0.04 (7.41 ± 0.71)	6.94 ± 0.09 (114.8 ± 26.5)	7.99 ± 0.05 (10.23 ± 1.25)	7.33 ± 0.07 (46.77 ± 8.18)

The calculated IC_50_ and EC_50_ values (in unit of nM) are listed in parentheses. The data are expressed as mean ± SE (N.D., not detectable).

Alternatively, we shortened the peptide length around B23–B25 position of R3/I5 by removing some residues. After B23Gly was removed, the resultant [∆B23]R3/I5 displayed ~300-fold lower activation potency towards both RXFP3 and RXFP4, although it retained considerable binding potency for both receptors (Figs 3A,B and 4A,B and Table 1). When both B23Gly and B24Gly were removed, the resultant [∆B23–24]R3/I5 lost all activation potency towards RXFP3, but the binding potency for this receptor was only ~3-fold lower than that of R3/I5 (Figs 3A and 4A and Table 1). Thus, [∆B23–24]R3/I5 was a strong antagonist for RXFP3. By contrast, [∆B23–24]R3/I5 exhibited ~1000-fold lower activation potency and ~10-fold lower binding potency towards RXFP4 compared with R3/I5 (Figs 3B and 4B and Table 1). Interestingly, further removal of B25Ser ameliorated the activity loss for both receptors: the resultant [∆B23–25]R3/I5 displayed ~30-fold lower activation potency and almost normal binding potency for both RXFP3 and RXFP4, compared with R3/I5 (Figs 3A,B and 4A,B and Table 1).

In summary, insertion or deletion in B23–B25 region had serious detrimental effects on the activation potency of R3/I5 for both RXFP3 and RXFP4 in most cases, although the binding potency for both receptors was largely unaffected. Thus, it seems that increasing the selectivity of R3/I5 for RXFP3 over RXFP4 cannot be achieved by changing the peptide length around this region.

### Substitution of B23Gly selectively abolishes the activation potency of R3/I5 towards RXFP3

When the highly conserved B23Gly was replaced by a slightly larger Ala or Ser residue, the resultant [G(B23)A]R3/I5 and [G(B23)S]R3/I5 lost all activation potency towards RXFP3, but both mutants retained considerable binding potency towards this receptor, especially [G(B23)A]R3/I5 (Figs 3C and 4C and Table 1). Thus, the B23 position appears to be an agonist/antagonist switch of R3/I5 for RXFP3: when occupied by a Gly residue, the peptide is an efficient agonist for RXFP3, but an Ala or Ser residue at this position results in a strong antagonist. By contrast, both [G(B23)A]R3/I5 and [G(B23)S]R3/I5 retained activation potency towards RXFP4, although they were ~100-fold less potent than R3/I5 (Fig. 4D and Table 1). However, both mutants retained high binding potency towards RXFP4, especially [G(B23)A]R3/I5 (Fig. 3D and Table 1).

In summary, replacement of B23Gly with Ala or Ser abolished the activation potency of R3/I5 towards RXFP3, but remained low activation potency towards RXFP4. Thus, it does not appear that increasing the selectivity of R3/I5 for RXFP3 over RXFP4 can be achieved by substitution of B23Gly.

### Substitution of B24Gly increases the selectivity of R3/I5 for RXFP3 over RXFP4

When the conserved B24Gly was replaced by a small Ala residue, the resultant [G(B24)A]R3/I5 not only retained full binding potency for both RXFP3 and RXFP4 (Fig. 3E,F and Table 1), but also retained full activation potency towards both receptors (Fig. 4E,F and Table 1). Thus, Ala replacement of B24Gly does not change the selectivity of R3/I5 towards RXFP3 and RXFP4.

When B24Gly was replaced by a Ser residue, the resultant [G(B24)S]R3/I5 also retained full activation and binding potencies towards RXFP3, compared with R3/I5 (Figs 3E and 4E and Table 1). However, this mutant displayed 20-fold lower activation potency and 2-fold lower binding potency towards RXFP4, compared with R3/I5 (Figs 3F and 4F and Table 1). As a result, [G(B24)S]R3/I5 displayed 20-fold higher activation potency towards RXFP3 than towards RXFP4 (Table 1). Since R3/I5 displayed almost equal activation potency towards RXFP3 and RXFP4, Ser replacement of B24Gly led to 20-fold increase of the selectivity of R3/I5 for RXFP3 over RXFP4, suggesting that the B24 position is a tuning site for enhancing the selectivity of R3/I5 towards RXFP3.

We also replaced B24Gly with Val, Thr, and Glu to test their effect on the receptor selectivity of R3/I5. When B24Gly was replaced by a hydrophobic Val residue, the resultant [G(B24)V]R3/I5 displayed ~10-fold higher activation potency towards RXFP3 than towards RXFP4 (Figs 3E,F and 4E,F and Table 1), suggesting that replacement with Val could also increase the selectivity of R3/I5 for RXFP3 over RXFP4. Unfortunately, this mutant displayed ~10-fold lower activation potency and ~5-fold lower binding potency for RXFP3, compared with R3/I5 (Figs 3E,F and 4E,F and Table 1), suggesting it is not a good RXFP3-selective agonist. When B24Gly was replaced by a Thr residue, the resultant [G(B24)T]R3/I5 displayed only ~2-fold higher activation potency towards RXFP3 than towards RXFP4 (Figs 3E,F and 4E,F and Table 1). Meanwhile, its activation potency for RXFP3 was ~20-fold lower than that of R3/I5, suggesting it is not a selective agonist for RXFP3. When B24Gly was replaced by the negatively charged Glu residue, the resultant [G(B24)E]R3/I5 lost all activation potency towards both RXFP3 and RXFP4 (Fig. 4E,F and Table 1), although it retained low binding potency towards both receptors (Fig. 3E,F and Table 1). In summary, it appears that [G(B24)S]R3/I5 is the best RXFP3-selective agonist obtained by replacement of B24Gly.

To further increase the selectivity of [G(B24)S]R3/I5 towards RXFP3, we tried introducing mutations at the conserved B25 position. When B25Ser was replaced by Ala, Gly, or Thr, the resultant double mutants all displayed over 500-fold lower activation potency for both RXFP3 and RXFP4, compared with R3/I5 (Fig. 4G,H and Table 1), although their binding potency for both receptors was largely unaffected (Fig. 3G,H and Table 1). Thus, B25Ser is important for the activation potency of R3/I5 towards both RXFP3 and RXFP4, and this position appears to be unusable for tuning the receptor selectivity of R3/I5.

In summary, substitution of B24Gly with Ser, Val, or Thr could increase the selectivity of R3/I5 towards RXFP3 over the homologous RXFP4. The best mutant, [G(B24)S]R3/I5, displayed 20-fold higher activation potency towards RXFP3 than towards RXFP4, meanwhile retained full activation potency at RXFP3 compared with wild-type R3/I5. To our knowledge, [G(B24)S]R3/I5 is the best RXFP3-selective agonist known to date. It is a valuable tool for studying the physiological functions of RXFP3, and also a suitable template for developing RXFP3-specific agonists in future.

## Discussion

In the present work, we developed a selective and fully active agonist for RXFP3, [G(B24)S]R3/I5, by extensive mutagenesis of the B-chain C-terminal region of the chimeric R3/I5 peptide. The B24Gly residue is highly conserved in relaxin-3s from different species (Fig. S1). In protein engineering experiments, highly conserved residues are typically not chosen in order to avoid disturbing protein function. However, our present work showed that the highly conserved B24Gly is completely tolerant of Ala or Ser replacement in terms of RXFP3 binding and activation. Thus, conserved residues can also be selected for protein engineering in some cases.

Generation of a specific agonist for RXFP3 has proved challenging for two reasons. The homologous RXFP4 has less stringent requirements for agonists compared with RXFP3, thus agonists for RXFP3 generally display high cross activity with RXFP4. Additionally, the three-dimensional structures of RXFP3 and RXFP4 are not yet available, thus the rational design of a specific agonist for RXFP3 is difficult or even impossible. As a result, trial-and-error methods must be employed that rely on a large number of mutant peptides. It is well known that relaxin family peptides are difficult to produce due to their complex primary structure (two polypeptide chains and three disulfide linkages). Although the separate A- and B-chains can be conveniently prepared by solid-phase peptide synthesis nowadays, combination of the synthetic chains is still challenging. In recent years, our laboratory established an efficient approach for producing mature relaxin family peptides based on overexpression of designed single-chain precursors in *E. coli* and subsequent *in vitro* refolding and enzymatic maturation^42–47^. Using this approach, mutant peptides can be quickly prepared at low cost. In the present work, we generated 14 R3/I5 mutants and obtained a selective and fully active agonist for RXFP3 from them.

The cognate agonist of RXFP3, relaxin-3, can also efficiently activate RXFP4 and RXFP1. In the brain, RXFP4 and RXFP1 are also expressed, thus a specific agonist will be helpful for elucidating RXFP3-mediated physiological functions. As the best RXFP3-selective agonist known to date, [G(B24)S]R3/I5 can serve this purpose in future studies. On the other hand, [G(B24)S]R3/I5 is also a suitable template for developing RXFP3-specific agonists. In future, we will try introducing mutation at other positions of [G(B24)S]R3/I5 in order to further improve its selectivity towards RXFP3.

## Methods

### Site-directed mutagenesis of R3/I5 and preparation of R3/I5 mutants

Site-directed mutagenesis of R3/I5 was conducted using the QuikChange method. The previously generated expression construct pET/R3I5 for overexpression of a single-chain R3/I5 precursor in *E. coli* was used as the mutagenesis template^42^. After the expected mutations were confirmed by DNA sequencing, mutant R3/I5 precursors were overexpressed in *E. coli* as inclusion bodies and solubilised through an S-sulfonation approach as previously described^38, 42^. S-sulfonated precursors were purified by immobilized metal ion affinity chromatography, and subjected to *in vitro* refolding as described in our previous studies^38, 42^. Refolded precursors were purified by HPLC using a semi-preparative C18 reverse-phase column (Zorbax 300SB-C18, 9.4 × 250 mm, Agilent Technology, Santa Clara, CA, USA), and then sequentially treated with endoproteinase Lys-C, papaya glutaminyl cyclase, and carboxypeptidase B, as described in our previous studies^38, 42^. Finally, mature R3/I5 mutants were purified by HPLC using an analytical C18 reverse-phase column (Zorbax 300SB-C18, 4.6 × 250 mm, Agilent Technology) and their identity confirmed by electrospray mass spectrometry on a QTRAP mass spectrometer (Applied Biosystems, Foster City, CA, USA).

### Circular dichroism spectroscopy

Mature R3/I5 mutants were dissolved in 1.0 mM aqueous hydrochloride solution (pH 3.0) and their concentrations were determined by absorbance at 280 nm using an extinction coefficient (ε_280nm_) of 7365 M^−1^·cm^−1^. Their final concentrations were adjusted to 20 μM in 1.0 mM aqueous hydrochloride solution (pH 3.0) for circular dichroism measurement, which was performed on a Jasco-815 spectrometer at room temperature. Spectra were scanned from 250 nm to 190 nm using a quartz cuvette with a 1.0 mm path length.

### Receptor binding assays

Receptor binding assays of the mature R3/I5 mutants were conducted using a NanoLuc-conjugated R3/I5 peptide as a tracer and human embryonic kidney (HEK) 293T cells transiently overexpressing human RXFP3 or human RXFP4 as a receptor source, as described previously^28, 38^. Briefly, HEK293T cells were transiently transfected with an expression construct of human RXFP3 or human RXFP4. On the next day, transfected cells were trypsinised, seeded into 96-well plates, and continuously cultured for 24–36 h to ~100% confluence. To initiate binding assays, medium was removed and binding solution (serum-free DMEM medium with 1% bovine serum albumin) containing 0.5 nM of the NanoLuc-conjugated R3/I5 tracer and various concentrations of competitor was added (100 μl/well). After incubation at 21 °C for 2 h, binding solution was removed and cells were washed twice with ice-cold phosphate-buffered saline (200 μl/well for each wash). Thereafter, cells were lysed by lysis solution (100 μl/well, Promega, Madison, WI, USA) and cell lysates were transferred to a white opaque 96-well plate (50 μl/well). After mixing with freshly diluted substrate (50 μl/well), bioluminescence was immediately measured on a SpectraMax M5 plate reader (Molecular Devices, Sunnyvale, CA, USA) in luminescence mode. Nonspecific binding was determined by competition with 1.0 μM wild-type R3/I5. The calculated specific binding data were expressed as means ± standard error (SE; *n* = 3) and fitted to sigmoidal curves using SigmaPlot 10.0 software.

### Receptor activation assays

Receptor activation assays of the mature R3/I5 mutants were conducted using the cAMP-response element (CRE)-controlled NanoLuc reporter as described previously^28, 38^. Briefly, HEK293T cells were transiently cotransfected with the NanoLuc reporter vector pNL1.2/CRE and the expression construct of human RXFP3 or human RXFP4. On the next day, transfected cells were trypsinised, seeded into 96-well plates, and continuously cultured for 24–36 h to ~90% confluence. To initiate activation assays, medium was removed and activation solution (serum-free DMEM medium plus 1% bovine serum albumin) containing 1.0 μM of forskolin and various concentrations of peptide was added (100 μl/well). After continuous culturing at 37 °C for 4 h, activation solution was removed and cells were lysed with lysis solution (100 μl/well, Promega). Cell lysates were then transferred to a white opaque 96-well plate (50 μl/well), mixed with freshly diluted substrate (50 μl/well), and bioluminescence was immediately measured on a SpectraMax M5 plate reader (Molecular Devices) in luminescence mode. The measured data were expressed as means ± SE (*n* = 3) and fitted to sigmoidal or linear curves using SigmaPlot 10.0 software.

## Electronic supplementary material

Supplementary Table 1 and Figure 1
