# Supplementary material for: Development of a selective agonist for relaxin family peptide receptor 3
Source: Sci Rep. 2017 Jun 12;7:3230. doi: 10.1038/s41598-017-03465-7 (PMC5468247; doi:10.1038/s41598-017-03465-7)
Supplement: Supplementary file 1 — Supplementary Table 1 and Figure 1 [file 41598_2017_3465_MOESM1_ESM.pdf]

**Supplementary information**

# Development of a selective agonist for relaxin family peptide receptor 3

Dian Wei[#], Meng-Jun Hu[#], Xiao-Xia Shao, Jia-Hui Wang, Wei-Han Nie, Ya-Li Liu, Zeng-Guang Xu, Zhan-Yun Guo*

Research Centre for Translational Medicine at East Hospital, College of Life Sciences and Technology, Tongji University, Shanghai, China

**Table S1. Molecular masses of the mature R3/I5 mutants measured by electrospray mass spectrometry.**

| Peptide | Measured mass | Theoretical mass |
|---|---|---|
| R3/I5 | 6169.0 | 6168.1 |
| [+G(B23-24)]R3/I5 | 6227.0 | 6225.1 |
| [ΔB23]R3/I5 | 6113.0 | 6111.0 |
| [ΔB23-24]R3/I5 | 6056.0 | 6054.0 |
| [ΔB23-25]R3/I5 | 5968.0 | 5966.9 |
| [G(B23)A]R3/I5 | 6183.0 | 6182.1 |
| [G(B23)S]R3/I5 | 6200.0 | 6198.1 |
| [G(B24)A]R3/I5 | 6183.0 | 6182.1 |
| [G(B24)S]R3/I5 | 6200.0 | 6198.1 |
| [G(B24)V]R3/I5 | 6211.0 | 6210.2 |
| [G(B24)T]R3/I5 | 6213.0 | 6212.1 |
| [G(B24)E]R3/I5 | 6242.0 | 6240.2 |
| [G(B24)S,S(B25)A]R3/I5 | 6181.0 | 6180.2 |
| [G(B24)S,S(B25)G]R3/I5 | 6169.0 | 6167.1 |
| [G(B24)S,S(B25)T]R3/I5 | 6211.0 | 6211.2 |

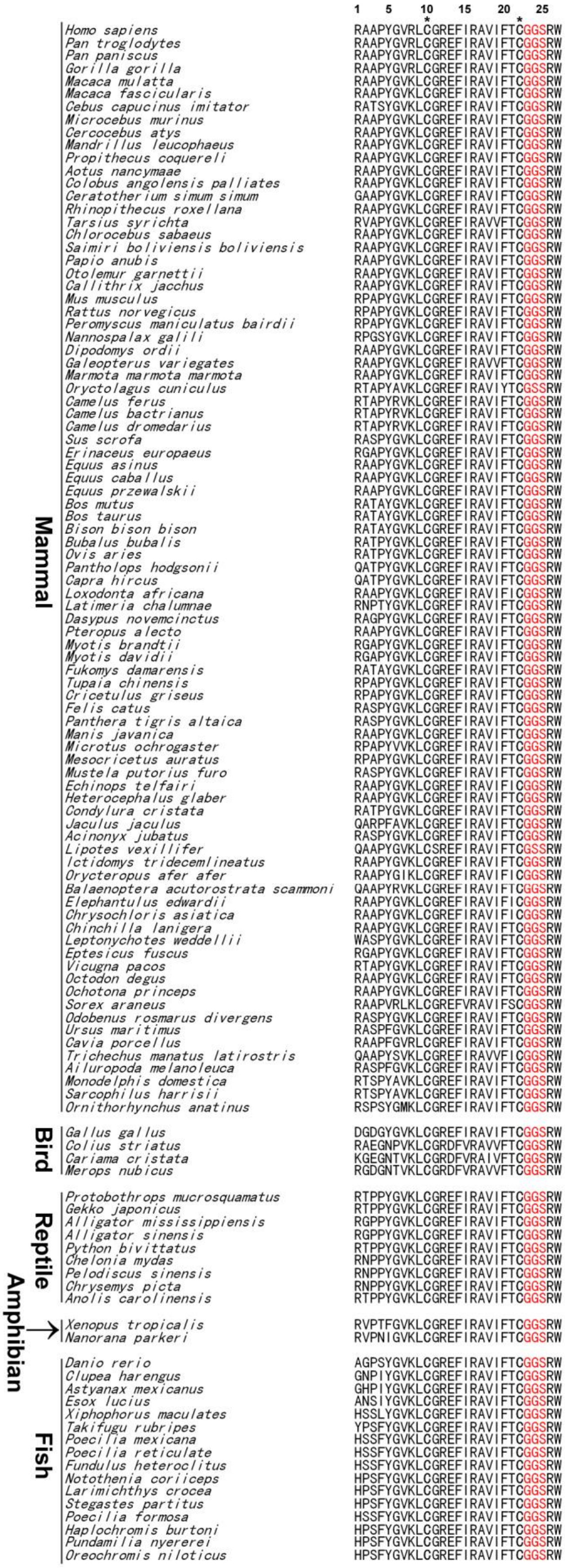
1 5 10 15 20 25
*
*
Mammal
Homo sapiens RAAPYGVRLCGREFIRAVIFTCGGSRW
Pan troglodytes RAAPYGVRLCGREFIRAVIFTCGGSRW
Pan paniscus RAAPYGVRLCGREFIRAVIFTCGGSRW
Gorilla gorilla RAAPYGVRLCGREFIRAVIFTCGGSRW
Macaca mulatta RAAPYGVKLCGREFIRAVIFTCGGSRW
Macaca fascicularis RAAPYGVKLCGREFIRAVIFTCGGSRW
Cebus capucinus imitator RATSYGVKLCGREFIRAVIFTCGGSRW
Microcebus murinus RAAPYGVKLCGREFIRAVIFTCGGSRW
Cercocebus atys RAAPYGVKLCGREFIRAVIFTCGGSRW
Mandrillus leucophaeus RAAPYGVKLCGREFIRAVIFTCGGSRW
Propithecus coquereli RAAPYGVKLCGREFIRAVIFTCGGSRW
Aotus nancymaae RAAPYGVKLCGREFIRAVIFTCGGSRW
Colobus angolensis palliates RAAPYGVKLCGREFIRAVIFTCGGSRW
Ceratotherium simum simum GAAPYGVKLCGREFIRAVIFTCGGSRW
Rhinopithecus roxellana RAAPYGVKLCGREFIRAVIFTCGGSRW
Tarsius syrichta RVAPYGVKLCGREFIRAVVFTCGGSRW
Chlorocebus sabaeus RAAPYGVKLCGREFIRAVIFTCGGSRW
Saimiri boliviensis boliviensis RAAPYGVKLCGREFIRAVIFTCGGSRW
Papio anubis RAAPYGVKLCGREFIRAVIFTCGGSRW
Otolemur garnettii RAAPYGVKLCGREFIRAVIFTCGGSRW
Callithrix jacchus RAAPYGVKLCGREFIRAVIFTCGGSRW
Mus musculus RPAPYGVKLCGREFIRAVIFTCGGSRW
Rattus norvegicus RPAPYGVKLCGREFIRAVIFTCGGSRW
Peromyscus maniculatus bairdii RPAPYGVKLCGREFIRAVIFTCGGSRW
Nannospalax galili RPGSYGVKLCGREFIRAVIFTCGGSRW
Dipodomys ordii RAAPYGVKLCGREFIRAVIFTCGGSRW
Galeopterus variegates RAAPYGVKLCGREFIRAVVFTCGGSRW
Marmota marmota marmota RAAPYGVKLCGREFIRAVIFTCGGSRW
Oryctolagus cuniculus RTAPYAVKLCGREFIRAVIYTCGSSRW
Camelus ferus RTAPYRVKLCGREFIRAVIFTCGGSRW
Camelus bactrianus RTAPYRVKLCGREFIRAVIFTCGGSRW
Camelus dromedarius RTAPYRVKLCGREFIRAVIFTCGGSRW
Sus scrofa RASPYGVKLCGREFIRAVIFTCGGSRW
Erinaceus europaeus RGAPYGVKLCGREFIRAVIFTCGGSRW
Equus asinus RAAPYGVKLCGREFIRAVIFTCGGSRW
Equus caballus RAAPYGVKLCGREFIRAVIFTCGGSRW
Equus przewalskii RAAPYGVKLCGREFIRAVIFTCGGSRW
Bos mutus RATAYGVKLCGREFIRAVIFTCGGSRW
Bos taurus RATAYGVKLCGREFIRAVIFTCGGSRW
Bison bison bison RATAYGVKLCGREFIRAVIFTCGGSRW
Bubalus bubalis RATPYGVKLCGREFIRAVIFTCGGSRW
Ovis aries RATPYGVKLCGREFIRAVIFTCGGSRW
Pantholops hodgsonii QATPYGVKLCGREFIRAVIFTCGGSRW
Capra hircus QATPYGVKLCGREFIRAVIFTCGGSRW
Loxodonta africana RAAPYGVKLCGREFIRAVIFICGGSRW
Latimeria chalumnae RNPTYGVKLCGREFIRAVIFTCGGSRW
Dasypus novemcinctus RAGPYGVKLCGREFIRAVIFTCGGSRW
Pteropus alecto RAAPYGVKLCGREFIRAVIFTCGGSRW
Myotis brandtii RGAPYGVKLCGREFIRAVIFTCGGSRW
Myotis davidii RGAPYGVKLCGREFIRAVIFTCGGSRW
Fukomys damarensis RATAYGVKLCGREFIRAVIFTCGGSRW
Tupaia chinensis RPAPYGVKLCGREFIRAVIFTCGGSRW
Cricetulus griseus RPAPYGVKLCGREFIRAVIFTCGGSRW
Felis catus RASPYGVKLCGREFIRAVIFTCGGSRW
Panthera tigris altaica RASPYGVKLCGREFIRAVIFTCGGSRW
Manis javanica RAAPYGVKLCGREFIRAVIFTCGGSRW
Microtus ochrogaster RPAPYVVKLCGREFIRAVIFTCGGSRW
Mesocricetus auratus RPAPYGVKLCGREFIRAVIFTCGGSRW
Mustela putorius furo RASPYGVKLCGREFIRAVIFTCGGSRW
Echinops telfairi RAAPYGVKLCGREFIRAVIFICGGSRW
Heterocephalus glaber RAAPYGVKLCGREFIRAVIFTCGGSRW
Condylura cristata RATPYGVKLCGREFIRAVIFTCGGSRW
Jaculus jaculus QARPFAVKLCGREFIRAVIFTCGGSRW
Acinonyx jubatus RASPYGVKLCGREFIRAVIFTCGGSRW
Lipotes vexillifer QAAPYGVKLCSREFIRAVIFTCGSSRW
Ictidomys tridecemlineatus RAAPYGVKLCGREFIRAVIFTCGGSRW
Orycteropus afer afer RAAPYGIKLCGREFIRAVIFICGGSRW
Balaenoptera acutorostrata scammoni QAAPYRVKLCGREFIRAVIFTCGGSRW
Elephantulus edwardii RAAPYGVKLCGREFIRAVIFICGGSRW
Chrysochloris asiatica RAAPYGVKLCGREFIRAVIFICGGSRW
Chinchilla lanigera RAAPYGVKLCGREFIRAVIFTCGGSRW
Leptonychotes weddellii WASPYGVKLCGREFIRAVIFTCGGSRW
Eptesicus fuscus RGAPYGVKLCGREFIRAVIFTCGGSRW
Vicugna pacos RTAPYGVKLCGREFIRAVIFTCGGSRW
Octodon degus RAAPYGVKLCGREFIRAVIFTCGGSRW
Ochotona princeps RAAPYGVKLCGREFIRAVIFTCGGSRW
Sorex araneus RAAPVRLKLCGREFVRAVIFSCGGSRW
Odobenus rosmarus divergens RASPYGVKLCGREFIRAVIFTCGGSRW
Ursus maritimus RASPFGVKLCGREFIRAVIFTCGGSRW
Cavia porcellus RAAPFGVRLCGREFIRAVIFTCGGSRW
Trichechus manatus latirostris QAAPYSVKLCGREFIRAVVFICGGSRW
Ailuropoda melanoleuca RASPFGVKLCGREFIRAVIFTCGGSRW
Monodelphis domestica RTSPYAVKLCGREFIRAVIFTCGGSRW
Sarcophilus harrisii RTSPYAVKLCGREFIRAVIFTCGGSRW
Ornithorhynchus anatinus RSPSYGMKLCGREFIRAVIFTCGGSRW
Bird
Gallus gallus DGDGYGVKLCGREFIRAVIFTCGGSRW
Colius striatus RAEGNPVKLCGRDFVRAVVFTCGGSRW
Cariama cristata KGEGNTVKLCGRDFVRAIVFTCGGSRW
Merops nubicus RGDGNTVKLCGRDFVRAVVFTCGGSRW
Reptile
Protobothrops mucrosquamatus RTPPYGVKLCGREFIRAVIFTCGGSRW
Gekko japonicus RTPPYGVKLCGREFIRAVIFTCGGSRW
Alligator mississippiensis RGPPYGVKLCGREFIRAVIFTCGGSRW
Alligator sinensis RGPPYGVKLCGREFIRAVIFTCGGSRW
Python bivittatus RTPPYGVKLCGREFIRAVIFTCGGSRW
Chelonia mydas RNPPYGVKLCGREFIRAVIFTCGGSRW
Pelodiscus sinensis RNPPYGVKLCGREFIRAVIFTCGGSRW
Chrysemys picta RNPPYGVKLCGREFIRAVIFTCGGSRW
Anolis carolinensis RTPPYGVKLCGREFIRAVIFTCGGSRW
Amphibian
Xenopus tropicalis RVPTFGVKLCGREFIRAVIFTCGGSRW
Nanorana parkeri RVPNIGVKLCGREFIRAVIFTCGGSRW
Fish
Danio rerio AGPSYGVKLCGREFIRAVIFTCGGSRW
Clupea harengus GNPIYGVKLCGREFIRAVIFTCGGSRW
Astyanax mexicanus GHPIYGVKLCGREFIRAVIFTCGGSRW
Esox lucius ANSIYGVKLCGREFIRAVIFTCGGSRW
Xiphophorus maculates HSSLYGVKLCGREFIRAVIFTCGGSRW
Takifugu rubripes YPSFYGVKLCGREFIRAVIFTCGGSRW
Poecilia mexicana HSSFYGVKLCGREFIRAVIFTCGGSRW
Poecilia reticulate HSSFYGVKLCGREFIRAVIFTCGGSRW
Fundulus heteroclitus HSSFYGVKLCGREFIRAVIFTCGGSRW
Notothenia coriiceps HPSFYGVKLCGREFIRAVIFTCGGSRW
Larimichthys crocea HPSFYGVKLCGREFIRAVIFTCGGSRW
Stegastes partitus HPSFYGVKLCGREFIRAVIFTCGGSRW
Poecilia formosa HSSFYGVKLCGREFIRAVIFTCGGSRW
Haplochromis burtoni HPSFYGVKLCGREFIRAVIFTCGGSRW
Pundamilia nyererei HPSFYGVKLCGREFIRAVIFTCGGSRW
Oreochromis niloticus HPSFYGVKLCGREFIRAVIFTCGGSRW

**Fig. S1.** Amino acid sequence alignment of the B-chain of relaxin-3s from different species. The two conserved Cys residues, that form interchain disulfide bonds with A-chain Cys residues, are shown in bold and indicated by asterisks. The B23–B25 region is shown in red.
